# Health system’s barriers hindering implementation of public-private partnership at the district level: a case study of partnership for improved reproductive and child health services provision in Tanzania

**DOI:** 10.1186/s12913-016-1831-6

**Published:** 2016-10-21

**Authors:** Denice Kamugumya, Jill Olivier

**Affiliations:** 1 University of Cape Town, School of Public Health and Family Medicine, Cape Town, South Africa; 2 University of Cape Town, School of Public Health and Family Medicine, Health Policy and Systems Division, Cape Town, South Africa

**Keywords:** Partnership, Engagement, Collaboration, Service level agreement, Non-state actors, Value, Contractual governance, Relational elements

## Abstract

**Background:**

Public-private partnership (PPP) has been suggested as a tool to assist governments in lower to middle income countries fulfil their responsibilities in the efficient delivery of health services. In Tanzania, although the idea of PPP has existed for many years in the health sector, there has been limited coordination, especially at a district level – which has contributed to limited health gains or systems strengthening obviously seen as a result of PPP.

**Methods:**

This case study was conducted in the Bagamoyo district of Tanzania, and employed in-depth interviews, document reviews, and observations methods. A stakeholder analysis was conducted to understand power distribution and the interests of local actors to engage non-state actors. In total 30 in-depth interviews were conducted with key informants that were identified from a stakeholder mapping activity. The initial data analysis guided further data collection in an iterative process. The provision of Reproductive and Child Health Services was used as a context. This study draws on the decision-space framework.

**Results:**

Study findings reveal several forms of informal partnerships, and the untapped potential of non-state actors. Lack of formal contractual agreements with private providers including facilities that receive subsidies from the government is argued to contribute to inappropriate distribution of risk and reward leading to moral hazards. Furthermore, findings highlight weak capacity of governing bodies to exercise oversight and sanctions, which is acerbated by weak accountability linkages and power differences. Disempowered Council Health Services Board, in relation to engaging non-state actors, is shown to impede PPP initiatives.

**Conclusion:**

Effective PPP policy implementation at a local level depends on the capacity of local government officials to make choices that would embrace relational elements dynamics in strategic plans. Orientation towards collaborative efforts that create value and enable its distribution is argued to facilitate healthy partnership, and in return, strengthen a district health system. This study highlights a need for new social contracts that will support integrative collaboration at the local level and bring all non-state actors to the centre of the district health system.

**Electronic supplementary material:**

The online version of this article (doi:10.1186/s12913-016-1831-6) contains supplementary material, which is available to authorized users.

## Background

### Introduction

Through its national public-private partnership (PPP) policy the Government of Tanzania recognises the important role of PPP in achieving social goals [1–4]. This policy was adopted in 2009 and later followed by the PPP Act in 2010 [5]. It stresses a need for the proper analysis of supporting structures (political, economic and social structures) to ensure there is appropriate distribution of risks and rewards between collaborating parties [6]. The Health Sector Strategic Plan III (2009-2015), further highlights the need to address gaps in health care delivery by avoiding overlaps and unnecessary competition with more emphasis on service level agreements (SLAs) [3]. This is also featured in the Health Sector Strategic Plan IV (2015-2020) [7]. SLAs are forms of contractual arrangements that provide opportunities for private providers to access public funding [3]. If well designed, SLAs are expected to address externalities, resource complementarity or recombination, and potential benefits gained between two entities leading to improved equity, efficiency and quality of services delivered [4, 8–11].

The increased recognition of PPP in the health sector is also seen in other emerging markets and large economies [12]. This trend is expected to grow globally as governments continue to face fiscal constraints in delivering social services [13–16]. The nature and scope of private providers differs from one country to the next, and the way in which their roles are incorporated into the national and sub-national health systems also vary [10, 17, 18]. For example, in high income countries, contractual governance mechanisms are seen to be more advanced, may constitute a large project (such as the private finance initiative in the United Kingdom), and tend to consider performance management measures and value creation elements. Several studies have explored the nature of such formal arrangements between collaborating parties [10, 12, 19]. In lower to middle income countries (LMICs), however, the contractual governance mechanisms are seen to be less developed. Relational governance, mostly with faith-based non-profit providers (which does not necessary take into account relational elements) is highlighted in the literature [8, 13]. The oversight of private providers is minimal, if any exist [16].

In Tanzania, at a district level, the private sector is highly heterogeneous, and consists of private not-for-profit (PNFP) providers such as faith-based providers and voluntary services, private for-profit (PFP) providers, local and international non-governmental organizations (NGOs), private practitioners operating alone, corporate private clinics and hospitals, traditional practice, informal and formal drug vendors, pharmacies, and stand-alone diagnostic laboratories [2]. Non-state actors provide a wide range of services: from preventive, to curative, to rehabilitative services - and in some areas are involved in pre-service training, pharmaceutical supply and construction and maintenance of facilities [4]. Facility-based PFP providers are more concentrated in urban and peri-urban areas [20–22]. PNFP providers, drug vendors, traditional practice and sporadically stand-alone diagnostic laboratories tend to operate in hard to reach remote areas [4, 23, 24].

There are a large number of PPP-related policy documents and reports available in Tanzania, but fewer empirical studies that show how these policy documents and reports are implemented. The available literature is also focused mainly on PPP at the national level. This study, using a qualitative lens, asks the following question: What are the governance-related health systems barriers that hinder implementation of PPP at a district level in Tanzania? The study intends to add knowledge on how to engage non-state actors, and in return contributes to a district health system’s strengthening approach.

### Conceptual background

Public-private partnership may take different arrangements between the government and private sector entities. The collaboration may involve renovation, construction, maintenance, management and provision of services in a whole or in part [6, 15, 18]. In other settings, outsourcing has been used as a modality to either tap resources from non-state actors (resource complementarity for service delivery) - when there are changes in the health care market conditions, or help a public entity shift its focus to a core business (service delivery) - by contracting out non-core business such as laundry, catering, housekeeping, and security services [18, 25].

Although, the idea of PPP has existed for several years in Tanzania, mixed performance is observed - influenced by sometimes ineffective governance mechanisms at the sub-national level [13]. It is further argued that the slow progress in improving overall health system’s performance can be ascribed to limited capacity to coordinate resources, identify effective technical strategy, and scale-up effective interventions, and inadequate managerial practices [26–28]. It has been highlighted that there is limited understanding and recognition of the concept of PPP at the district level [4, 24]. Furthermore, missed opportunities to introduce SLAs in areas where some population groups are underserved have been highlighted [4]. For example, in other locations private providers are the only facilities operating in a given geographical area, but for those who are entitled to exemptions, fee exemptions do not apply. This is also seen when public facilities have stock-outs of essential medicines, which happens frequently [21, 22, 28]. This indicates the need for effective mechanisms to provide services based on need, and not ability to pay, by working together with all types of providers.

Widely promoted reforms in retail services in the last decade are seen to contribute to dispersed Accredited Drug Dispensing Outlets (ADDOs), also known as essential drug shops. Although these reforms were initiated to address the rural and peri-urban local need for pharmaceutical and medical supplies, they seem to bring in new relational dynamics that require effective governance mechanisms [23]. The same trend is seen with stand-alone private diagnostic laboratories [4]. Gaps have been highlighted with these reforms, for example lack of quality assurance leading to poor quality of services, and uncontrolled price of commodities, which exacerbates inequity [4, 21, 28]. Such reforms could lead to collaboration, competition, and conflict among involved entities in the political arena, which may further contribute to system underperformance [29].

The revised District Health Management Information System (DHMIS) provides an opportunity to use reliable data to make evidence-based decisions that can inform contractual negotiations and performance management with non-state actors [30–32]. With the potential for information asymmetry however, this would depend on how non-state actors are engaged at the district level, and relationships established with shared values [33]. It is argued that in a long-term partnership formal contracts and trust, when combined, may provide a desired policy outcome [34]. The decentralization offers another opportunity to engage non-state actors in planning and policy dialogue at the local level [35]. This is also because local authorities are more familiar with who non-state actors are in their areas, and could easily monitor their performance, and improve systems performance [16, 36, 37].

There is strong PPP technical coordination at the national level in Tanzania as demonstrated by the existence of the PPP Unit under the Ministry of Health and Social Welfare (MoHSW), PPP-Technical Working Group, and various PPP dialogues [1, 4]. At the district level, however, the strong coordination would depend on the district health system’s governance supported by appropriate competencies to effect change. This includes the ability to facilitate inclusive policy dialogue and strategic plans at the local level that would improve the accountability and decision-making processes in order to achieve health goals [35, 37]. It is further appreciated that governance is a ‘key mechanism permitting value creation and shape its distribution’ [11].

**Fig. 1:**
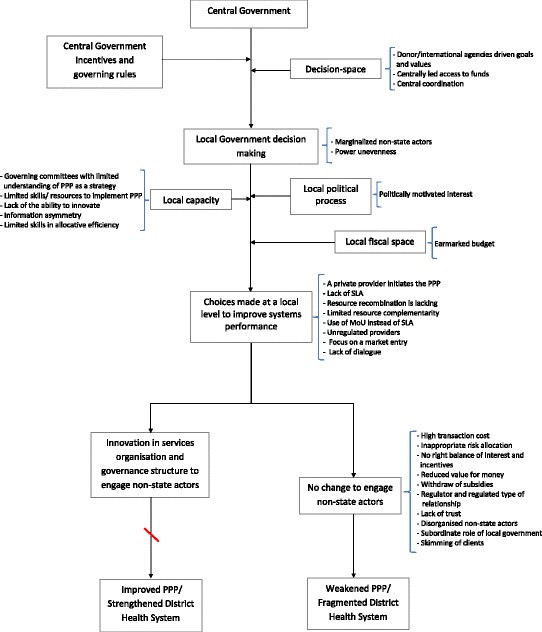
Study framework and key findings guided by the decision-space framework [35]. The study design is guided by this framework, with a focus on decentralization. Key findings (important sub-themes) are provided that corespond to a respective domain on the framework

This study draws on the decision-space framework (Fig. 1), which considers a decision-space as the amount of choices (degree of autonomy) transferred to local officials in a decentralized system [35]. This study focused on the decision-space in service organisations more broadly, and on contracting with private providers in particular. The characteristics of local actors in PPP, and the type of choices local officials (as agents) can make to improve system performance through PPP were also explored. The local fiscal space is considered, but how finances and other incentives are allocated from the central government-the principal, and PPP central governing rules as defined by law were not within the scope of this study.

## Methods

A case study approach was employed to gain an understanding of multiple interconnected factors underpinning PPP [38, 39]. Bagamoyo district, the district of focus in this study, in the Eastern zone of Tanzania is situated 75 km North of Dar-es-Salaam, the main business and trading city in the country. This district has an urban-rural population presenting appropriate district for this case study. Bagamoyo district was selected for the study primarily on the basis of research access and relevance to the topic.

Bagamoyo district has one hospital, which is publicly owned, while private facilities operate at levels of health centres and dispensaries [40]. This presents a difference with other districts in Tanzania, which often have faith-based appointed district hospitals known as Council Designated Hospitals. However, with the decentralized system, the governance structure is the same across all districts in Tanzania. The policy leadership and stewardship role is provided by the MoHSW, and the operational role is undertaken by local government [3].

Since PPP involves multiple implementing partners, provision of Reproductive and Child Health Services (RCHS) was used as the context or as a tracer to uncover the socio-political complexity in PPP policy implementation [41]. Based on MoHSW guidelines RCHS are considered as essential services, some services are required to be provided by private providers free of charge, and in the retail sector there is a fee and services may be provided without doctor’s consultation [4, 42]. The focus on RCHS at a district level was expected to provide the complexity expected in PPP, and simultaneously make this study manageable.

The sample of stakeholders for this study was purposefully selected from a stakeholder map (see Additional file 1) that was established after a stakeholder analysis. The stakeholder analysis was conducted to understand power distribution and the interests of local actors to engage non-state actors. Consideration was given to the two parliamentary constituencies in the district (Bagamoyo and Chalinze), in order to get a proper representation of non-state actors, given political influence that shapes actors relations [41]. To ensure all levels at the district were represented, two divisions from each constituency were purposefully selected, followed by one ward from each selected division and one village from each selected ward. The selection was based on the scale of RCH initiatives in those areas (large scale and small-scale initiatives) and was done through document review that included monthly, quarterly and annual reports, and the Comprehensive Council Health Plan (CCHP), as well as initial interviews with key informants.

Data collection employed in-depth interviews with key informants, observations of participants, and document review methods. For governing committees at different levels in the district, either the chairman or secretary participated in interview. For non-state actors, owners or managers of a facility or particular service were interviewed. This included dispensaries, health centres, maternity home, pharmacies, essential drug shops, ADDOs, NGOs, research institute, and private company through Corporate Social Responsibility. The saturation point was reached with 30 interviews in total. A social network sketch (see Additional file 2) was used for each interview as a tool to facilitate identification of actors’ linkages or interactions – including establishing the capacity to supply information or respond to/ impose sanction (Table 1). To ascertain whether PPP mechanisms that do exist are successful (effective) the focus was on how these mechanisms have contributed to improved equity in accessing care (access based on need, not ability to pay), availability of essential medicines, and quality of care. Written consent was obtained for interviews (with voice recording in most cases) and when conducting observations. All scripts were coded, and no personal identifiers were used. Field notes were used to collect observation data, focusing on physical interactions and discussions between private providers and service users. Document reviews and observations assisted to identify other RCHS providers, and establish if there were SLAs that exist within the local district authority, how the SLAs were initiated, and the type of the agreement (to determine how answerability and sanction are taken into account).

Recorded interviews were transcribed verbatim in Swahili and then translated to English. This was then followed by manual coding, organising and reorganising data and emerging themes. The initial analysis guided further data collection in an iterative process. Data from each category of non-state actors (Table 1) were analysed and triangulated with observational and documentary review data separately. Themes were generated for each category, and then interrelated across categories to identify emerging patterns. Reflexivity was considered to manage authors’ influence on the findings. Respondent validation was instituted to ensure meaning was not distorted.

## Results

In this case study a number of important themes emerged that include factors that hinder and support PPP. In this article, we report mainly on factors that hinder the implementation of the PPP policy in Bagamoyo district in Tanzania. Findings are summarized in Fig. 1.

### Public-private partnership and governing bodies: Challenges and limitations

There are health governing organs at all three levels in the district. The District Council Team, Council Social Services Committee, Council Health Services Board (CHSB) and Council Health Management Team (CHMT) at the district level, the Ward Development Committee, and Ward Health Committee at the ward level, and the Village Health Committee, which was also referred by community members as Facility Health Committee for a respective public dispensary in the village.

Based on available guidelines, at the district level, the higher governing body with the private sector representation is the CHSB. However, in Bagamoyo District, the CHSB is not strong as private sector representation lacks authenticity. This is seen from responses from facility managers and owners of ADDO, pharmacies and stand-alone diagnostic laboratories. As one participant said:

*“I have heard of the CHSB, but I don’t know what is usually discussed. We don’t have a representative. We (private providers) are considering forming an association and then appoint leaders, who would then be our representatives. But we have not yet managed to form that association” (Participant 21 – Bagamoyo 2014).*

Members of CHSB are endorsed by the Ward Development Committee, after demonstrating interest to be members, and then assessed by the CHMT, before being recommended to the District Executive Director for appointment. However, one of the ward officials noted that the endorsement of non-state actors’ representatives to CHSB does not take into account the ability of an individual to mobilize others to promote public interest:

*“There was one person from this ward who completed the application form for board’s membership, and we (ward officials) did approve it…Those positions (to be a CHSB member) are advertised and forms are usually made available at ward offices…It is very difficult to forward negative comments…The only time we can deny an individual this position is when that person is mentally not normal” (Participant 20 – Bagamoyo 2014).*

Although the CHMT is very strong as it has personnel with strong biomedical skills and other resources to their disposal, they are more inclined towards public facilities. This is acerbated by the public distrust of the private sector, as a significant number of those who operate private practice are government employees. This was the response from one of key informants:

*“The relationship with private providers is not well perceived by the public. There is a perception that they (public and private staff) favour each other. The main argument is items are embezzled from the public facilities, and get sold in private facilities” (Participant 23 - Bagamoyo 2014).*

The District Council Team is very strong and makes executive decisions; however, it is more inclined towards the public than the private sector. As a result the Council Team directs the CHMT and CHSB, which also has some CHMT members as board members, to focus on public facilities. The Council Team focuses more on how resources are channelled to public facilities in order to win voters’ popularity rather than on strategies that would improve equity, efficiency and quality of care. The CHSB has the potential to foster the engagement of non-state actors, however the CHSB is weak as it lacks resources for effective PPP implementation:

*“The big role of the CHSB is to control procurement and distribution of drugs and other medical supplies at public facilities. The linkage between other committees and the CHSB is on how public complaints are conceived at lower levels and how they get channelled to the CHSB” (Participant 25 - Bagamoyo 2014).*

There are wards where the Ward Health Committees do not exist, and in other wards the Ward Health Committees do exist but are weak in terms of the PPP policy implementation as they have limited understanding of PPP as a concept or strategy:

*“I would say our committee (Ward Health Committee) is non-functional, completely non-functional since last year. We tried to revive it but there were complaints among members about general skills development” (Participant 20 – Bagamoyo 2014).*

Village (Public Facility) Health Committees have limited scope and their main focus is on public facilities. Like all other committees, Village (Public Facility) Health Committees have limited capacity to demand information from non-state actors, and exercise oversight and sanctions:

*“There are a lot of issues that we don’t know. We are only two of us (me and the in-charge of the public facility in the village) who attended training at the district, and it was a brief session. You cannot grasp everything in such a short training” (Participant 24 – Bagamoyo 2014).*

### Strategic decisions to improve responsiveness: Limited inclusion of actors in planning and decision-making

It was revealed by key informants that the CHMT prepares the Comprehensive Council Health Plan (CCHP) annually that has to be endorsed by the District Council Team before being approved by the Prime Minister‘s Office Regional Administration and Local Government (PMO-RALG) and the MoHSW. All relevant policy guidelines are provided by the MoHSW. However, it was observed in this research that the private sector is not adequately represented in strategic decisions. The CHSB is not directly involved in strategic planning. Those whose names appear in the CCHP document as representatives of non-state actors did not actually participate in strategic sessions, reflecting inadequate inclusion of all services providers. These were some responses from individuals whose name appears on the CCHP document as a representative of non-state actors:

*“I used to attend strategic meetings back in early 2000s as a representative of (non-state actors) but after I took another job I stopped. So if you tell me I am still a representative, that is not right, I don’t attend those meetings…” (Participant 1 – Bagamoyo 2014).*

“*I usually hear about it (CCHP). I have never seen a copy…I don’t understand why my name should appear in that document as a representative” (Participant 9 – Bagamoyo 2014).*

The local government is able to promote the Community Health Fund (CHF) membership (a voluntary pre-payment scheme), as directed by the Ministry’s guidelines. Room to improve physical access by engaging non-state actors is not well explored at the local level. This is compounded by the requirement for matching subsidization from the central government. In one of the visited villages, the NFP provider is easily accessible and well equipped. Nevertheless, CHF members who also include secondary school girls cannot use that facility as CHF is restricted to public facilities. Members are required to walk a considerable distance to access care. When medicines are out-of-stock, which is the case in most public facilities in this district, they are required to pay cash at private facilities or ADDOs:

*“We (CHMT and CHSB) can make our decisions at the district level after receiving guidelines from the ministry however, for CHF members to receive services from private providers we need to plan it well. We have to get them (private providers) sensitized. For now we have no options” (Participant 17 – Bagamoyo 2014).*

Regarding district health data, data available from DHMIS, developed at the national level, are analysed and discussed during the CHMT meetings. However, it was demonstrated by one informant that collected data is of poor quality, or not digestible or actionable to improve PPP at the district level [30].

*“In one activity you may find you have twenty indicators. For example under DHMIS there are about eight reports. At the same time there are other reports required for each section such as RCHS, PPP, etc… this becomes an additional workload. There is no way you can get a good quality report” (Participant 18 – Bagamoyo 2014).*

It was further highlighted by another informant that collected data is required by the Ministry, which reflects lack of innovation to use available data to address emerging challenges at the local level. For example, reports from one facility-based private provider indicate a high workload that does not match the number of staff available. However, little has been done to address this issue despite the fact that this facility receives subsidies from the government:

*“The government needs to find a way to support us. They (government) see our monthly reports. In a month, a number of under-fives attended here is between 1000 and 2000, new cases of pregnant mothers is between 70 and 80, leave alone ‘re-attendance’. For family planning, we report 200 clients. That is a very tough job…We really struggle to get staff. And I understand even at government facilities there is a shortage, but there are ways we can work together on this” (Participant 21 – Bagamoyo 2014).*

**Table 1:**
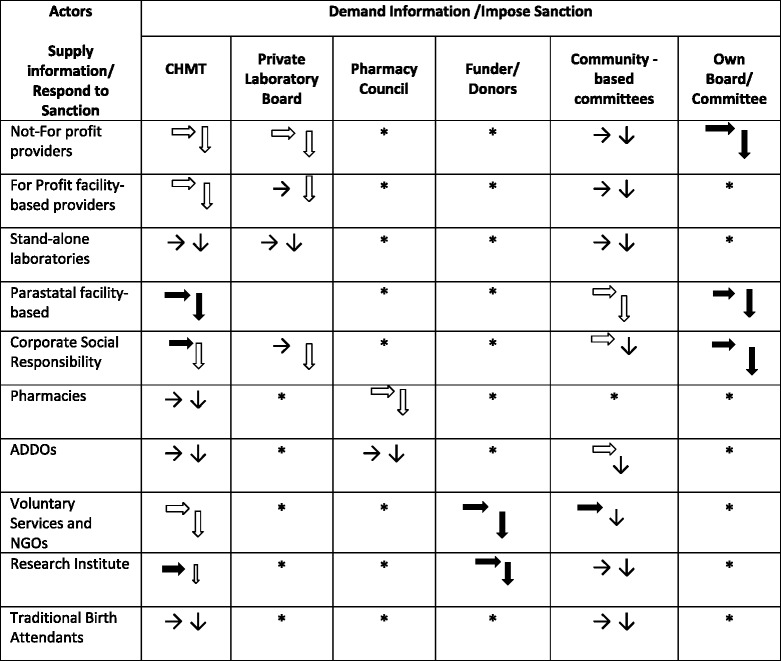
PPP actors’ accountability matrix [44]

* No accountability linkages among actors

This was established using a social network sketch, where the interviewee was asked to map relational (formal and informal) linkages, and then explain the nature of the relationship. The capacity to supply information or responded to sanction was analysed based on the ability of the respective provider to submit reports or complying with directive from the authority or another party and consequence thereof: Weak →; Medium ; Strong

Likewise, the capacity to demand information or impose sanction was established based on how the authority or another party is empowered in this regard and how it exercises power: Weak ↓; Medium ; Strong

Limited inclusion of actors in planning and decision-making is also reflected in how logistics are managed. There is a lack of innovation to facilitate change as seen with Integrated Logistics System for supplies that come from the Medical Store Department as a fixed stock. There is limited flexibility to ensure that stock is constantly maintained and an alternative source is easily established with other local suppliers such as ADDO or other facility-based providers through PPP, when there is a shortage of supplies at public facilities:

*“There are times you may receive the new stock from the Medical Store Department but since it is a fixed stock you may be in a situation where there is an increased need for a particular treatment. In such a situation it becomes very difficult to approach the company (the partner) for assistance…” (Participant 30 – Bagamoyo 2014).*

### Performance accountability: Poor performance of accountability structures

The CCHP does outline health outcomes that need to be collectively achieved in the district. However, there is a weak accountability linkage structure among actors (Table 1), which is limited to supplying information creating a room for non-compliance. More emphasis is given to individual service’s entry to the market through registration and licencing [43]. The feedback provided to facility-based providers usually focus on gaps in quality of data collected and areas for improvement in data collection only. It is observed that a platform for explanation and justification, leading to dialogue between the two parties has not been created [44].

*“There is no feedback provided once we have submitted our reports. We don’t discuss anything further. When you see the performance of Bagamoyo District as a whole, you just assume you were part of the success/ failure…and it is only when you are lucky to attend higher level meetings” (Participant 3 – Bagamoyo 2014).*

Drugs shops and pharmacies provide family planning pills and some sell injectable contraceptives, but performance accountability in this aspect does not fall under the district RCHS or PPP focal person. There is a separate reporting system that has been created for ADDOs and private pharmacies by the Pharmacy Councils - a regulatory body, which is centrally coordinated. However, a lot of inconsistency of who complies and who does not was observed. There is no evidence of the reporting feeding back to the district from the Pharmacy Council. For stand-alone private diagnostic laboratories, most of them are not registered and do not submit reports anywhere (Table 1).

*“We are accountable to the pharmacy council, as we submit our monthly reports to them…things like family planning are included. So they get that information straight from us…and how they use it is up to them” (Participant 12 – Bagamoyo 2014).*

### Contractual governance: Lack of service level agreements

It was mentioned by one informant that in order to comply with the requirement of establishing formal partnerships, the district is in the process of putting into place Memoranda of Understanding (MoUs) with facilities that receive subsidies from the government. Another informant mentioned that programs that are not funded through the local government, such as those implemented by NGOs and research project-oriented type of collaborations, usually have contracts in the form of an MoU.

*“A General MoU does exist… The partnership is more research project-oriented…a laboratory was constructed for the project… the scope of collaboration is oriented towards the fund committed to the project” (Participant 6 – Bagamoyo 2014).*

Nevertheless, the district has not signed SLAs with any non-state actor, including those who receive subsidies from the government (Table 2).

*“There are those services/ programs with contracts, for example those who get funds from outside the country… Programs that are funded by the local government usually do not have contracts” (Participant 23 – Bagamoyo 2014).*

This lack of SLAs poses an inappropriate distribution of risks and rewards, limiting mechanisms that have the potential to promote public goals. For example, it was mentioned by a key informant that the district hospital occasionally gets free supplies from private providers such as NFPs when they run out-of-stock, but NFP providers buy supplies from wholesalers, at a higher price when compared to the Medical Store Department. In such a situation NFP providers may adjust to cope with this new demand, and leave value-driven social services [36].

**Table 2:** Types of partnerships with the local government that do exist in the district

Type of Providers	Type of Contractual Agreement	Type of Collaboration
Faith-Based Provider	None	Provision of RCHS but excludes family planning. Supplies are provided free of charge, and staff are seconded from local government. In return services offered are free of charge.
Faith-Based provider	None	Informal arrangements at village level for staff availability.
Faith-Based provider	None	Informal arrangements between public facilities, and the private provider such as transfer of vaccines from one facility to the other during power blackout.
PFP and PNFP providers	None	Informal arrangements for assistance when a public facility runs out-of-stock such as for syringes, gloves, etc. Such assistance is usually free of charge but at times a replacement has to be sent at a later stage. Some private providers receive reagents for Voluntary Counselling and Testing (VCT) services.
Private pharmacies and faith-based providers	General Contract	Contractual arrangements between the National Health Insurance Fund (NHIF) and private providers, but restricted to pharmacies and faith-based providers. ADDO and private for profit are not part of providers’ network.
Maternity home	None	Various forms of PPP arrangements with the maternity home such as outreach- point for immunization, free of charge supplies for some RCHS including Prevention of Mother to Child Transmission of HIV (PMTCT) however, services are not entirely free, clients have to contribute and the contribution is determined by the provider.
Jointly operated facility, private estate company and government	None	The company provided a building, house for seconded staff, employ some staff, and procure and maintain a stock for its employees, while the government provides, supplies through its Integrated Logistics System for the community, and overall oversight of the facility, and second staff. RCHS are provided as per government guidelines.
Traditional Birth Attendant (TBAs)	None	There are some of facilities that have introduced incentives for TBAs who facilitate referrals of pregnant mothers for facility delivery.
NGOs/ Private company	MoU	Partnership with NGOs (at local or national levels) in construction of staff houses, renovation of facilities, sexual and reproductive health initiatives, and HIV/AIDS prevention care and treatment initiatives.
Parastatal-based facilities	None	Parastatal-based facilities now operating like public facilities. Initially they had their own arrangements managed through their respective Ministerial headquarters.
Research Institute	MoU	Research project-oriented collaborations. The partnership is initiated at a time when the project is commissioned, and ends at the end of the project. It may involve construction and renovation of buildings, operating, and then transfer.
Out sourcing	None	In case of out-of-stock at the Medical Store Department. The district procurement officer would purchase a new stock from the appointed contractor, though the contractor tends to change each year.

*“Occasionally, I have supported the district hospital with drugs when they run out of stock… We are not getting anything from the Medical Store Department (MSD). We buy our supplies from a wholesaler, but issue them free of charge. We also give to the district hospital for free” (Participant 3 – Bagamoyo 2014).*

It was noted that a lack of written agreement at the service level contributes to misunderstandings between parties, and there are service interruptions that are experienced as a result. This was the case with Prevention of Mother to Child Transmission of HIV (PMTCT) service provision where private providers receive subsidized Antiretroviral (ARV) drugs and reagents from the government. In order to receive these supplies private providers have to submit their reports monthly, something that is not done consistently. This has created an unhealthy relationship between parties as there are those government staff who strictly adhere to this requirement, and others who issue ARV drugs and reagents without adhering to this requirement:

*“With private providers, there is a challenge as there are those who provide incomplete data, and those who do not respond. For example data on HIV, we need that information as they (private providers) get subsidized reagents from the government, but you will find that they (private providers) provide incomplete information claiming that they buy other items using their own resources” (Participant 8 – Bagamoyo 2014).*

Faith-based providers are faced with limited resources, and are constrained against offering the services that they would prefer. In addition, they now charge for services, which they would prefer not to charge. This is the case with National Health Insurance Fund (NHIF) contracts where members are requested to pay in cash when NHIF delays the reimbursement.

*“…because we do not receive supplies from the government, it is a problem. There are situations when we get stuck, and we notify our clients/patients (including those under NHIF) to contribute… It is difficult to run a facility if you buy something at TSH 20,000/- and be expected to sell it at TSH 3,000/-…” (Participant 29 – Bagamoyo 2014).*

In another facility that is in the process of establishing RCHS, it was mentioned that:

*“We are building (additional buildings for new RCHS) slowly, as the revenue that we get we have to distribute it to salaries, buying medical supplies and drugs, as well as food for staff. We are doing it slowly” (Participant 27 – Bagamoyo 2014).*

Additionally, the in-charge of one of the NFP providers indicated that the faith-based facility cannot start providing RCHS if the government would not provide staff. It was also noted that the interpretation of PPP is limited to sharing staff – government seconding staff to private providers.

*“I have heard of service level agreement. We did discuss this in this process of establishing RCHS, because we wanted staff to be seconded here, and we were ready to provide housing and allowances” (Participant 16 – Bagamoyo 2014).*

## Discussion

Much effort is being made to promote PPP and the private sector at the district level in Bagamoyo. However, findings from this study highlight that these efforts are not well-coordinated, and effective mechanisms have not been established as yet to tap resources from or for the private sector. Non-state actors are seen to be severely marginalized in district strategic planning.

The presence of non-state actor representatives’ names in the district strategic planning documents that are not authentically represented, brings to light the characteristics of actors who govern the decision-making process. As argued by ‘public choice’ theorists, this reveals the self-interest driven behaviour of local government officials and the use of ‘power as thought control’ to influence decisions of executive meeting at the district and national levels [45]. Core elements for PPP such as inclusion, transparent, and ethical behaviours remain under-recognised [46].

It has been highlighted that faith-based providers are often involved in policy dialogue, as they are seen to have a common goal with the government [9, 13, 36, 47]. Findings from this study, however, indicate NFP providers are also not involved in decision-making at the district level. Although faith-based providers are relatively well organised in Tanzania [16], and have strong representation in policy dialogues at the national level through their umbrella organisation (The Christian Social Services Commission) [4], in this case study they are weakly represented at a district level.

Local governments are expected to have more decision-making power on how funds are spent and with what type of provider [4]. However, this study reveals uneven power distribution between governing bodies at the district level. The CHMT is equipped with strong personnel with biomedical skills, and other resources, but perceives PPP narrowly. The District Council Team is powerful and tends to influence decisions made by CHMT and CHSB, however it is inclined to place politically motivated interests over value distribution, which has been argued to affect the engagement of non-state actors [11]. Study findings highlight limited skills on allocative efficiencies given competing priorities to improve systems performance through PPP.

Findings from this study highlight many weak performance accountability linkages. The capacity to demand information (supply information) and institute (respond to) sanction and regulatory oversight are expected to bridge the gap in the information asymmetry between the principal-local government and agent-private providers [37, 44]. Study findings here indicate a wide gap in information sharing as the relationship that is seen is that of ‘regulator and regulated type’ [16]. Relational elements dynamics such as commitment to shared values, high level of trust and interactive problem-solving are not perceived as centrally important by the local government officials in district planning, and are exacerbated by the lack of dialogue with non-state actors. It has been highlighted that relational elements, when they complement other governance mechanisms, are likely to yield desirable outcome [31].

Other studies in Tanzania and Zimbabwe found that regulation of private providers is oriented towards individual services rather than health systems organisation and/or market-level challenges [43]. Similar findings are seen in Bagamoyo district on how ADDO shops, private pharmacies, and stand-alone private diagnostic laboratories are regulated by bodies which do not have a direct link to the office of the District Medical Officer or CHMT - further fragmenting the system. Such governance arrangements, which are less integrative, tend to give private actors strong authority, and managerial discretion that come at increased cost of supervision [31]. It has been highlighted that engaging the private sector, in the form of information exchange and in planning may yield better results - rather than regulation alone [48].

Findings from this study reveal different forms of collaboration between the local government and the private sector. However, partnerships with non-state actors are informal with the exception of those not funded through the local government, which have MoUs in place. Furthermore, it has been argued that PPP as a concept is narrowly perceived at the local level [24] which is also demonstrated in this study. However, the current strategy by this district to introduce MoUs for non-state actors who receive government subsides reflects how policies are usually re-shaped and re-interpreted in unexpected ways by front-line bureaucrats thus directly affecting policy outcomes [26, 49, 50]. Contractual relations (such as MoUs) that provide for more flexibility require on-going analysis of contractual dynamics, of social processes that promote trust, solidarity and information exchange, in a context with a potential market failure [34, 51]. This kind of effective coordination is lacking in Bagamoyo District.

Conversely, formal contractual arrangements such as SLAs, are expected to address every future contingency including the intended quality of services [11, 34]. The absence of written contractual agreements with current partnerships in this district highlights how collaboration with non-state actors is usually initiated at a local level. It is argued that such partnerships do not take into account the proper analysis of political, economic and social structures [6]. A lack of reliable and consistent data to make informed decisions during PPP negotiations and further performance management affect value creation [31]. When non-state actors initiate partnership with the local government (the case for most current and forming collaborations in Bagamoyo District), such collaborations tend to lose public interest as a result. As argued in the application of agency theory [37, 52], and supported by these findings, the local government may not have enough capacity to monitor non-state actors, who may decide to pursue their own interest. The local public sector turns out to be in a sub-ordinate role in such a situation, due to information asymmetry [10, 12].

Well-designed SLAs can improve risk allocation and achieve value for money through reducing environmental uncertainties and compensating for market externalities [9–11]. On the other hand, the lack of a SLA may impede providers’ behaviour that favours public interests [37]. This study reveals non-state actors’ ‘moral hazards’ as a result of inappropriate distribution of risk and rewards. For example, restrictions on who should receive services based on their ability to pay, and not on need, by introducing cost sharing mechanism for RCHS that would have been provided partly free of charge. Correspondingly, some facilities are avoiding types of collaborations that would require provision of RCHS. This is contributed to by the failure to achieve the right balance of interests and incentives [46]. For example, high transaction costs for setup and operation of a facility has been highlighted to contribute to the inability to achieve intended policy outcome [10].

It has been further highlighted that the extension of PPP to a wider range of services requires consideration on how incentives are aligned against administrative complexity [10] - as such extension tends to come with increased transaction cost [11, 34]. In this study, restricted flexibility in spending is seen to deter prompt actions to address evolving population need such as collaborating with private providers to establish new RCHS, and improve physical access to care for CHF members. This can also be attributed to a narrow local fiscal space, as more than 95 % of the local total health expenditure is earmarked once the CCHP has been approved by the central government. Since this study focused on service organisation at the district level, further studies could usefully look into central incentives and governing rules to promote PPP at local levels.

## Conclusion

PPP continues to offer promising results, however limited capacity of local government to make choices that would improve health outcomes is highlighted as an inhibiting factor of the national efforts to promote PPP. Provision of public services by private providers without formal contractual agreements limits the progress towards attaining health goals through PPP. Uneven power between stakeholders impedes inclusiveness of the private sector in district strategic planning. A higher governing body at the district level (that is expected to have the private sector representation) is not empowered to oversee other governing bodies in strategic decision-making. As more efforts continue to be put into promoting the private sector, local governments need to orient themselves towards collaborative efforts that create value and enable its distribution rather than just licensing and regulation.

This case study provides insights on how to strengthen policy implementation at the sub-national level, and strengthen the district health systems as a result of PPP. As the role of the private sector in contributing to public social goals is increasingly gaining recognition in LMICs, this study underscores a need for a type of social arrangement that is underpinned by shared values and embedded in a broader society and economy [53]. These types of social arrangements should support centrally coordinated national PPP initiatives, and facilitate integrative collaboration at the local level that forms a basis for governance decisions.

## Additional files

Additional file 1: Public-Private Partnership Policy Actor Map – September 2014. This map shows all actors in the district, and their position as related to PPP. (PDF 111 kb)
Additional file 2: A social network sketch. This is a tool that was used to establish accountability linkages. (PDF 77 kb)

## Abbreviations

ADDO(s): Accredited Drug Dispensing Outlet(s)
ARV: Antiretroviral
CCHP: Comprehensive Council Health Plan
CHF: Community Health Fund
CHMT: Council Health Management Team
CHSB: Council Health Services Board
DHMIS: District Health Management Information Systems
LMICs: Lower to Middle Income Countries
MoHSW: Ministry of Health and Social Welfare
MoU: Memorandum of Understanding
NGO(s): Non-Governmental Organisation(s)
NHIF: National Health Insurance Fund
PFP: Private for Profit
PMO-RALG: Prime Minister Office – Regional Administration and Local Government
PMTCT: Prevention of Mother to Child Transmission of HIV
PNFP: Private Not for Profit
PPP(s): Public-Private Partnership(s)
RCHS: Reproductive and Child Health Services
SLA(s): Service Level Agreement(s)
TBA(s): Traditional Birth Attendant(s)
