# Supplementary figures and images for: Health system’s barriers hindering implementation of public-private partnership at the district level: a case study of partnership for improved reproductive and child health services provision in Tanzania
Source: BMC Health Serv Res. 2016 Oct 21;16:596. doi: 10.1186/s12913-016-1831-6 (PMC5073970; doi:10.1186/s12913-016-1831-6)

Public-Private Partnership Policy ACTOR MAP – September 2014

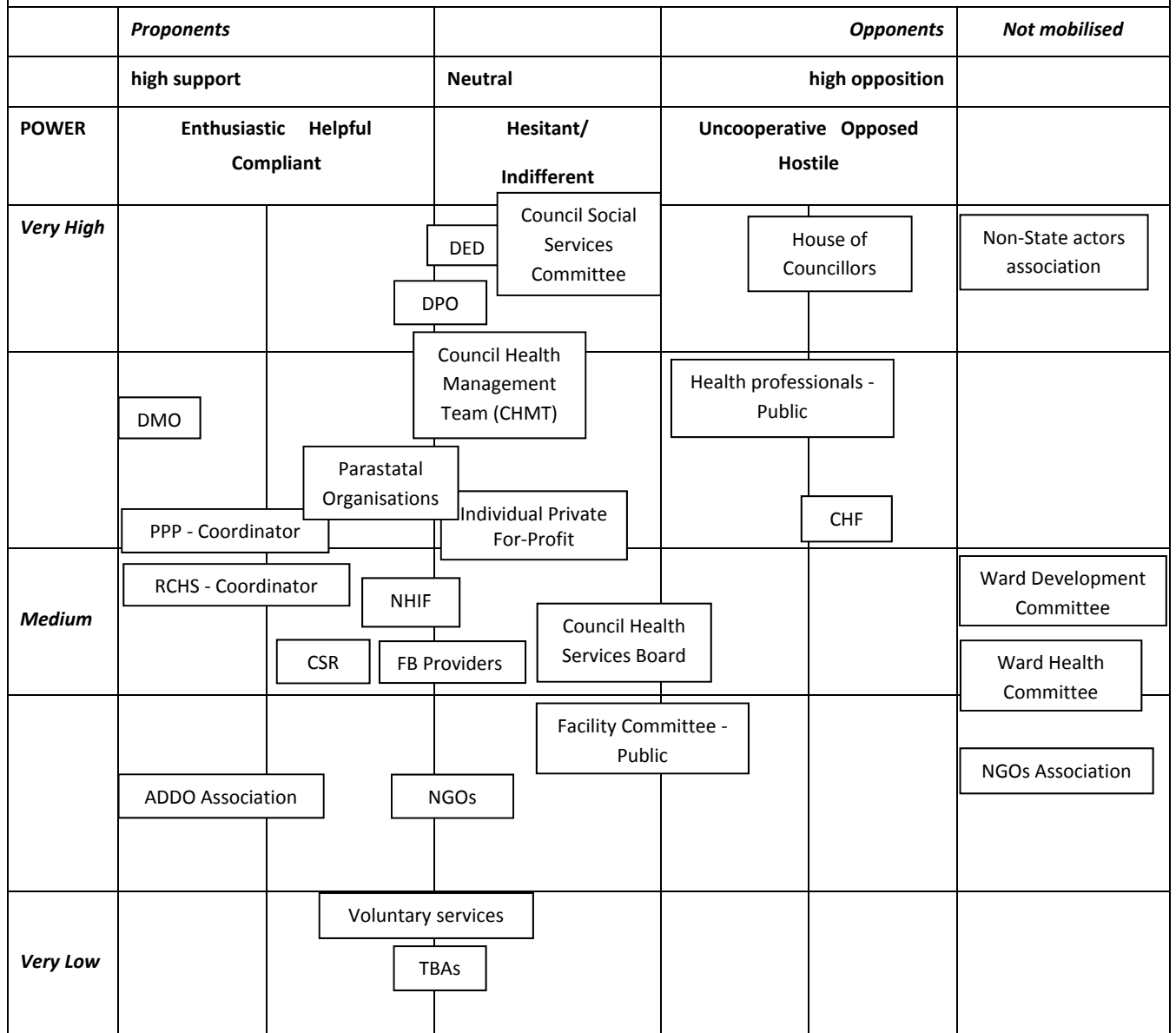

Supplement: Additional file 1: — Public-Private Partnership Policy Actor Map – September 2014. This map shows all actors in the district, and their position as related to PPP. (PDF 111 kb) [file 12913_2016_1831_MOESM1_ESM.pdf]
