# Supplementary material for: Health system’s barriers hindering implementation of public-private partnership at the district level: a case study of partnership for improved reproductive and child health services provision in Tanzania
Source: BMC Health Serv Res. 2016 Oct 21;16:596. doi: 10.1186/s12913-016-1831-6 (PMC5073970; doi:10.1186/s12913-016-1831-6)
Supplement: Additional file 2: — A social network sketch. This is a tool that was used to establish accountability linkages. (PDF 77 kb) [file 12913_2016_1831_MOESM2_ESM.pdf]

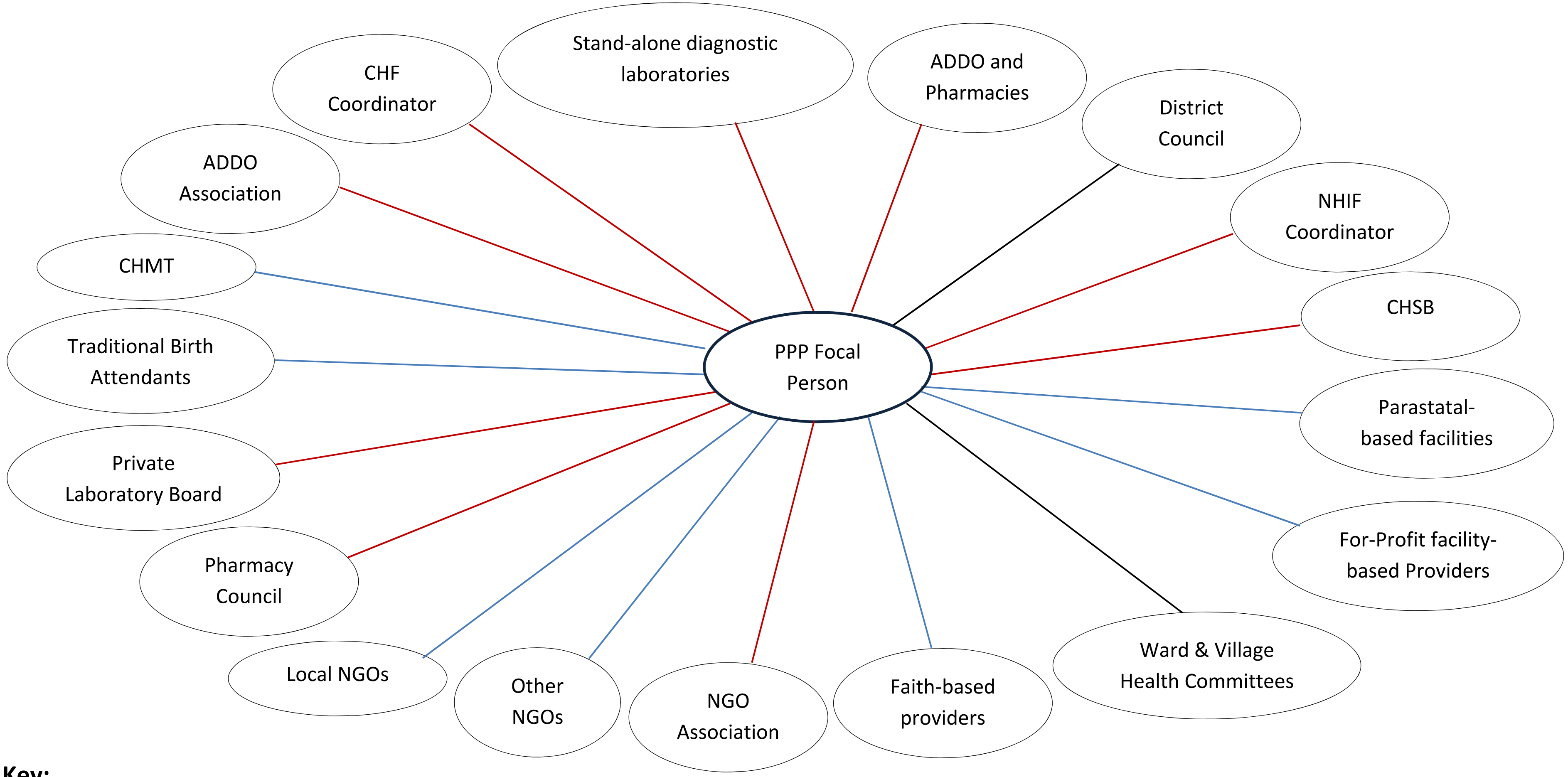

Key:

Fairly good relationship

Some improvement required

Opportunity for a relationship (none currently exist)
